# Perceptual–Cognitive Function and Unplanned Athletic Movement Task Performance: A Systematic Review

**DOI:** 10.3390/ijerph17207481

**Published:** 2020-10-14

**Authors:** Jan Wilke, David Groneberg, Winfried Banzer, Florian Giesche

**Affiliations:** 1 Department of Sports Medicine and Exercise Physiology, Goethe University Frankfurt, 60488 Frankfurt/Main, Germany; 2 Division of Preventive and Sports Medicine, Institute of Occupational, Social and Environmental Medicine, Goethe University Frankfurt, 60590 Frankfurt/Main, Germany; groneberg@med.uni-frankfurt.de (D.G.); banzer@med.uni-frankfurt.de (W.B.); giesche@sport.uni-frankfurt.de (F.G.)

**Keywords:** unanticipated, decision-making, brain function, sports, athletes, cognition

## Abstract

The performance of choice-reaction tasks during athletic movement has been demonstrated to evoke unfavorable biomechanics in the lower limb. However, the mechanism of this observation is unknown. We conducted a systematic review examining the association between (1) the biomechanical and functional safety of unplanned sports-related movements (e.g., jumps/runs with a spontaneously indicated landing leg/cutting direction) and (2) markers of perceptual–cognitive function (PCF). A literature search in three databases (PubMed, ScienceDirect and Google Scholar) identified five relevant articles. The study quality, rated by means of a modified Downs and Black checklist, was moderate to high (average: 13/16 points). Four of five papers, in at least one parameter, found either an association of PCF with task safety or significantly reduced task safety in low vs. high PCF performers. However, as (a) the outcomes, populations and statistical methods of the included trials were highly heterogeneous and (b) only two out of five studies had an adequate control condition (pre-planned movement task), the evidence was classified as conflicting. In summary, PCF may represent a factor affecting injury risk and performance during unplanned sports-related movements, but future research strengthening the evidence for this association is warranted.

## 1. Introduction

During the last decades, interactive sports have experienced a variety of changes, in essence, becoming faster and more dynamic [1]. Between 1966 and 2010, ball speed in football (soccer) increased by 15 percent, while the passing rate rose by 35 percent [2]. Additionally, in a recent seven-year interval (2006 to 2012), the number of high-intensity actions per game doubled [1]. Similar observations were made in other sports. For instance, an analysis of the average men’s single tennis first serve velocity during the French Open tournament showed a continuous upward trend from 160 km/h in 1991 to 188 km/h in 2009 [3]. These data impressively reflect the growing demands on athletes, which do not only include peripheral factors such as strength and power but also cognitive abilities.

Individuals in most sports are required to process a multitude of external (e.g., opponents, teammates, and ball) and internal (own position and joint stability) stimuli, constantly adapting their own motor actions within seconds or milliseconds [4]. A variety of skills, commonly referred to as markers of perceptual–cognitive function (PCF), seem paramount for time-constrained decision-making. As an example, a football player needs visual scanning to rapidly screen the environment, short-term/working memory to memorize and interpolate opponents’ and team mates’ positions/movements, and inhibitory control/cognitive flexibility to stop, modify or switch motor actions depending on the dynamics of the situation (e.g., a change in direction to sidestep a defender). It has been suggested that high levels of PCF may help to increase sports performance, while individuals with lower levels may even be at increased injury risk because no timely and/or adequate reaction can be initiated in response to contextual threats such as an imminent collision with another athlete [5].

Despite the arguably high relevance of PCF in sports, the majority of the applied diagnostic screening methods lack significant cognitive demands. Frequently, assessments of strength, range of motion or balance rely on controlled, pre-planned single-task movements with limited time pressures, which arguably reduces ecological validity. Indeed, Teramoto et al. [6] found that conventional parameters such as strength or power had no or only small predictive values for game performance in basketball. Furthermore, the detected variance explanations of motor outcomes were lower than those of non-modifiable length-size variables such as height or wingspan. Similar findings were obtained by Viscovi et al. [7]. After examining ice hockey players participating in pre-season screenings of the American National Hockey League (NHL), the authors concluded that no single off-ice test was related to on-ice performance. 

Researchers have attempted to increase test contextuality and ecological validity by means of combining a typical athletic motor task with a time-constrained decision-making component. Besier et al. [7] instructed their participants to perform straight runs towards a screen, which spontaneously displayed the direction of an immediate cutting maneuver. Interestingly, these unplanned side cuts were associated with unfavorable knee biomechanics when compared to a pre-planned task indicating the cutting direction already before the run. The findings of Besier et al. [8] are in line with a later systematic review of experimental trials, showing that unplanned movements lead to changes in lower limb kinetics and kinematics, which are suggestive of an increased injury risk [9]. 

Recently, Giesche et al. [10] and Niederer et al. [11] found that indicating the required landing leg not before but during a jump causes a substantial number of erroneous task executions. Generally, performing an inadequate reactive motor action may trigger injury (e.g., if subsequently colliding with an opponent) or compromise performance (e.g., if subsequently losing the ball). Athletic task safety can hence be decreased not only from a biomechanical but also from a functional point of view.

Although it is plausible that the ability to perform unplanned cutting/landing tasks under time pressures may be dependent on PCF, this question has not been examined systematically, hitherto. The present review summarizes the available evidence regarding the association between PCF and markers of biomechanical and functional task safety during unplanned athletic movement. 

## 2. Materials and Methods

A systematic literature review was performed in July and August 2020. It was conducted adhering to the PRISMA (Preferred Reporting Items for Systematic Reviews and Meta-Analyses) guidelines [12] and followed the recommendations for the ethical publishing of systematic reviews proposed by Wager and Wiffen [13]. The study was registered in the PROSPERO database (CRD42018089914).

### 2.1. Search Strategy

Two independent investigators (Jan Wilke and Florian Giesche) performed a systematic literature search. In a first step, relevant articles, published without date restrictions, were identified by means of the online databases MEDLINE (PubMed) and ScienceDirect. The search term used was “(neurocognition OR cognition) AND (unanticipated OR unplanned OR choice-reaction) AND (cutting OR landing)”. In addition, the reference lists of all papers relevant to the research question were checked [14]. As the omission of unpublished data has been shown to potentially bias the results of reviews [15], additional searches were performed with Google Scholar, screening the first 100 hits obtained with the above term. 

### 2.2. Inclusion Criteria

Experimental trials examining markers of (1) PCF and (2) biomechanical (e.g., joint moments, ground reaction force or postural sway) or functional (e.g., correct decision-making) safety during unplanned movement tasks were included. Papers had to be written in the English language and published in peer-reviewed journals. Published PhD theses (indexed in Google Scholar) meeting the criteria were also included and highlighted accordingly. 

### 2.3. Data Extraction

Using a standardized assessment sheet, two investigators (Jan Wilke and Florian Giesche) independently performed the data extraction. The following information was retrieved: participant characteristics, movement tasks, PCF and biomechanical outcomes and results (Table 1). If trials reported PCF and biomechanical/functional markers of task safety but did not examine potential relationships between them (e.g., by means of correlation analyses or inference statistics), we requested the raw data from the corresponding authors in order to perform the respective calculations.

### 2.4. Study Quality and Synthesis of Evidence

Two examiners (Jan Wilke and Florian Giesche) independently rated study quality by means of an adapted version of the Downs and Black checklist [16]. It has been proven as a feasible and reliable tool for assessing the methodological characteristics of randomized and non-randomized studies [16,17]. Our modified instrument (Table 2) included a maximum number of 16 items grouped into four categories: reporting quality, external validity, internal validity (risk of bias), and power. For each criterion met, 1 point was awarded, and a sum score (maximum, 16 points) was calculated. 

The recommendations of the Cochrane Collaboration Back Review Group [18] were applied to rate the available evidence as strong (consistent findings of multiple high-quality studies), moderate (consistent findings among multiple low-quality studies and/or one high-quality study), conflicting (inconsistent findings among multiple studies) or not existent (no studies available).

## 3. Results

### 3.1. Search Results

**Figure 1:**
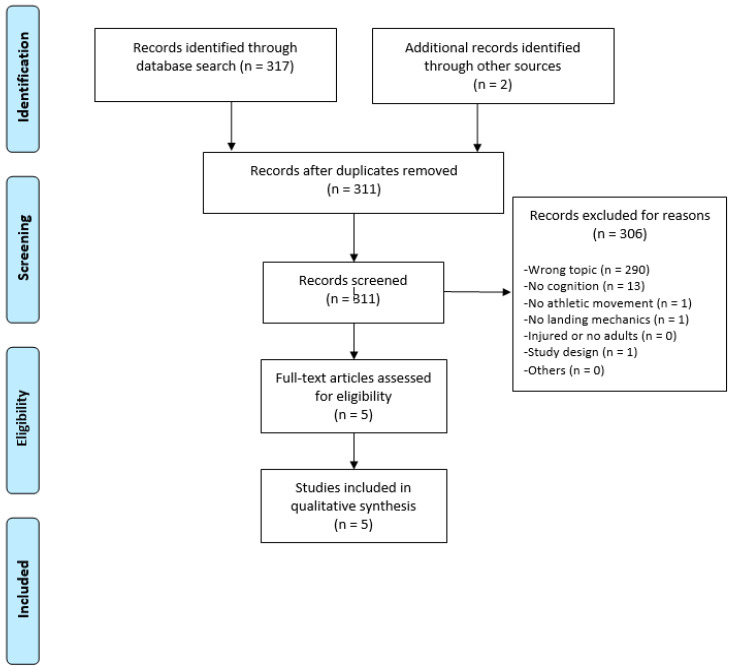
Chart displaying the literature search.

The literature search (Figure 1) returned 421 potentially relevant studies. After the removal of duplicates and application of exclusion criteria, four cross-sectional studies [10,19,20,21] and one crossover study [11] were included (Table 1). Raw data had to be requested from the authors of one paper [11]. 

### 3.2. Methodological Quality and Risk of Bias

Methodological quality was generally high (mean: 13/16 points), ranging from 11 to 15 points (Table 3). With regard to sub-scores, the values were largest for reporting (minimum: 8/9 points). By contrast, none of the studies linked the objective with a target population, and hence, all received 0 points for participant representativeness. Three out of the five studies did not adjust for relevant confounders (e.g., jump height/running speed, stimulus duration, and available reaction time), and similarly, three out of five studies had potentially insufficient power. 

### 3.3. Individual Study Findings

The experimental set-up of Herman and Barth [19] required an initial jump from a 30 cm box onto a force plate. Shortly (250 ms) before landing, a second target (frontal left, frontal right or the same force plate), which had to be reached with an immediate rebound, was indicated on a screen. No pre-planned control condition was completed by the recreationally active participants. The authors divided their sample into a low (LP) and high (HP) cognitive performer group using a computer-based assessment of reaction time, visual scanning and object recognition (Concussion Resolution Index). The LP group produced 31% higher peak vertical ground reaction forces, 26% higher tibial anterior shear forces and 15-fold higher knee abduction moments. Furthermore, the LP participants landed with greater knee abduction (6.1° ± 4.7° vs. 1.3° ± 5.6°) and smaller trunk flexion angles (9.6° ± 9.6° vs. 16.4° vs. 11.2°).

Shibata et al. [20] let female university elite athletes perform single-leg (dominant limb) drop-jump landings from a 30 cm box onto a force plate, which were followed by one of three maneuvers: a side cut, the maintenance of the single-leg stance, or a forward step. Again, only unplanned trials were performed, and the participants were grouped into HP and LP based on a pen-and-paper test (Symbol Digit Modality test), which captures psychomotor speed, visual short-term memory, attention and concentration. No differences in hip and knee peak joint angles, joint moments and electromyographic hamstring muscle activity were found. However, the LP group exhibited higher quadricep activity 50 ms before (+93%) and in the 50 ms after the initial ground contact (+70%). This resulted in a smaller hamstring-to-quadricep co-contraction ratio, both pre- (−63%) and post-initial contact (−45%).

In the study of Almonroeder [21], females (physically active, at least on a recreational level) jumped onto a force plate to complete either a single-leg landing, a bilateral landing with a vertical jump or a single-leg landing with a lateral cut. Both pre-planned (required landing maneuver indicated prior to the jump) and unplanned trials (indication during flight) were examined. Only the side cutting on the non-dominant limb during the first 100 milliseconds after initial contact was analyzed. Participants were categorized into LP/FP using the computer-based ImPACT test battery capturing cognitive processing speed. No significant group x condition interactions were found, either for knee and hip angles and knee moments or for stance time (the latency between the landing and cutting). However, there was a significant group main effect, indicating that the LP group demonstrated significantly higher ground reaction forces in both the pre-planned (+17%) and unplanned conditions (+20%).

Giesche et al. [10] instructed male recreational athletes to perform counter-movements on a pressure plate. The required landing leg was indicated either prior to the jump (pre-planned) or during the flight phase (unplanned). The authors examined the correlations between PCF (multiple computer and pen-and-paper-based tests) and (1) unplanned biomechanical landing costs (differences between planned and unanticipated landings) as well as (2) landing (using the wrong or both legs)/standing (inability to maintain a stable stance after landing) errors. With regard to postural sway (center of pressure path length), unplanned landing costs correlated with lower interference control (Stroop word-color interference test; r = 0.48). Furthermore, significant relationships between an increased number of unplanned landing errors and lower working memory/cognitive flexibility (Trail-Making Test; TMT-B, r = 0.54; TMT-B vs. A, r = 0.47) as well as short-term memory (Digit Span test, r = 0.55) were found. Finally, the number of standing errors correlated with better working memory/cognitive flexibility (TMT-B, r = 0.48) and short-term memory (Digit Span test, r = 0.50). 

Niederer et al. [11] investigated the acute effects of different warm-up interventions and neuromuscular fatigue on single-leg landing biomechanics, landing success (landing/standing errors) and cognitive function (TMT-A and B, capturing visual-perception working memory/cognitive flexibility) using a randomized controlled crossover design. The experimental set-up was identical to the study of Giesche et al. [9], but only unplanned tasks were performed. Our statistical analyses of the original data sent by the authors (control warm-up/pre-fatigue condition) revealed a significant relationship between the number of landing errors (defined as landing on the wrong side or an inability to maintain a stable stance after ground contact) and lower visual scanning (TMT-A; r = 0.7). The landing biomechanics (the time to stabilization and peak ground reaction force) did not correlate with PCF, although a non-significant trend (*p* < 1) for a possible association between higher peak ground reaction forces and lower cognitive flexibility (TMT-B vs. A difference, r = 0.45) was identified. 

### 3.4. Synthesis of the Available Evidence

The ratings of the available evidence, stratified for the used outcomes, are displayed in Table 4. Evidence was classified as conflicting for both the associations between PCF and biomechanical task safety, and PCF and functional task safety during unplanned athletic movement (inconsistent findings from multiple high-quality studies).

## 4. Discussion

Although a large body of evidence has revealed that the performance of unplanned sports-related movement patterns is associated with biomechanical aberrations suggestive of increased injury risk [9], there is still a scarcity of studies investigating the underlying mechanism. Our review identified only five studies addressing the potential link between (a) PCF and (b) changes in biomechanics and functional task safety during cutting or landing tasks requiring time-constrained decision-making. Evidence from the available papers is conflicting.

When seen from a global perspective, with the exception of the thesis from Almonroeder [21], all the analyzed studies, in at least one parameter, indicated a possible impact of PCF on landing or cutting safety. High levels of PCF may enable athletes to make rapid and accurate decisions under high time constraints. Compared to individuals with lower PCF, such accelerated cognitive processing of external stimuli arguably provides additional time to correct inadequate movements and plan upcoming motor actions. Coaches wanting to increase sports performance may hence consider implementing screenings and exercises requiring time-constrained decision-making during sports-related movement. The potential association between PCF and unplanned movement safety could also be relevant from an injury-preventive perspective. Faster and more-precise decision-making may enlarge the time frame for producing feedforward muscle activity, which is considered crucial to ensure joint stability after ground contact, e.g., following a jump [22]. Interestingly, initial evidence indeed indicates that lower baseline (pre-season) PCF predisposes to non-contact injuries of the lower limb [23,24,25].

However, when considering the findings of our review in more detail, several aspects call for further research. Firstly, besides the participant characteristics (e.g., sex and sports expertise level), the applied movement tasks (e.g., cuts vs. drop-jumps and counter-movement jumps), the chosen outcomes (functional vs. biomechanical parameters of task safety; batteries vs. single tests for PCF) and the statistical analyses performed were highly heterogeneous between studies. The study authors, furthermore, in the majority of the cases, did not specify clear null hypotheses, performed a substantial number of significance tests without controlling for alpha error inflation and frequently failed to include 95% confidence intervals and effect sizes. Taken together, all this makes generalizations of the data difficult. Secondly, only two [10,21] out of five studies compared pre-planned and unplanned movement tasks. While the correlations between lower PCF and unfavorable biomechanics or inadequate motor actions during unplanned movements are intriguing findings, they could only be interpreted as proof of causality if a similar association does not exist in pre-planned trials. In other words, future studies examining PCF and unplanned task safety require an adequate control condition (i.e., pre-planned movement task). Thirdly, the studies included in this review mainly tested lower-order cognitive skills, which describe basal information processing (e.g., psychomotor speed, reaction time or visual scanning [26]). In football (soccer), a typical interactive sport, executive function, which is an example of higher-order skills allowing complex decision-making and task switching (e.g., involving working memory, cognitive flexibility and inhibitory control [27,28]), has been identified as a predictor of performance [29,30,31]. Interestingly, while higher-order skills seem to discriminate elite athletes from amateur and recreational athletes, no difference between the populations can be found for lower-order PCF [31]. As jump landings and cuts represent typical movement patterns in football, we argue that biomechanical and functional task safety under unplanned circumstances requiring time-constrained decision-making will particularly depend on higher-order functions. Finally, the studies included in this review followed the indirect perception paradigm. It assumes a “top-down” approach in which external stimuli (a) are converted into meaningful representations based on experience and expectation, (b) cognitively processed and (c) used for the initiation of an appropriate motor response. However, in view of the limited storage capacity of the brain and the manifold degrees of freedom in movement, this concept has been criticized [32,33]. Direct perception models are based on a “bottom-up” approach suggesting that the environment offers countless possibilities for action (“affordances”), which are immediately registered by and potentially acted upon by the receiver without further internal information processing. Future studies, therefore and in summary, (1) require testing against different theoretical frameworks, (2) clearly specified hypotheses, (2) contextually valid outcomes and (3) adequate pre-planned control conditions.

## 5. Conclusions

The findings of the present systematic review provide initial evidence for a potential link between PCF biomechanics and functional task safety during cutting or landing tasks requiring time-constrained decision-making. However, the results need to be interpreted with caution. Although the methodological quality of the included trials was generally high, evidence from the available papers is conflicting and leading to potentially ambiguous conclusions, which seems partly due to methodological aspects. Future studies considering the implementation of pre-planned control conditions and higher-order cognitive testing are warranted. 

## Figures and Tables

**Table 1:** Characteristics of the included studies.

Study	Participants	Movement Task	Outcomes	Statistics
Herman [19]	*n* = 37 recreational athletes (HP: *n* = 20; 10 males; LP: *n* = 17; 8 males), age: 21 years; height: 1.72 m; weight: 68.9 kg; cross-sectional design	*Unanticipated drop-jump landing* Forward jump from 30 cm box onto force plate (placed at a distance measuring one-half of the participant’s height) with immediate rebound (bilateral) to a second target (left, right or vertical; visual cue indicating target position displayed before landing on first force plate) - Use of dominant limb - Available response time: minimum of 250 ms - Visual stimuli: arrow sign (left, right or straight) - Stimulus location: in front of participants - Floor/target visible: yes	*Biomechanics:* 3D kinematic and kinetic data of the dominant limb and trunk *Cognitive function:* computerized test (CRI) to capture simple and complex reaction time/processing speed, visual scanning	Participants subdivided into HP (average CRI percentile, 78th) and LP (average CRI percentile, 41st) group based on total score of cognitive testing; between-group differences (biomechanics)
Shibata [20]	15 healthy female athletes (age: 20 ± 1 years, BMI: 22 kg/m^2^), jumping/cutting sports in university athletic clubs at highest national competition level (2–3 training hours daily, 5–6 days/week); cross-sectional design	*Unanticipated single-leg landing with side-cutting* Drop-jump (30 cm box) on force plate with subsequent maneuvers, side-step cutting 45° (CUT), single-leg landing (LAND) or forward stepping (STEP), arrow indicating the required movement task after leaving the box, displayed on screen; only CUT analyzed - Use of dominant limb - Available response time: minimum of 240 ms (fall time) - Visual stimulus: color symbols (yellow horizontal arrow: cut, blue circle: land, red upward arrow: step) - Stimulus location: 4 m in front of participants (30 cm above ground) - Floor/target visible: yes	3D kinematic and kinetic data (dominant limb), averaged muscle activity (%MVC): HAM, QUAD, CCR (ratio of QUAD:HAM (recorded pre-/post-IC)) *Cognitive function:* pen-and-paper test (SDMT) to capture psychomotor speed, visual short-term memory, attention and concentration	Participants subdivided into HP vs. LP group based on total score of cognitive testing (median); between-group differences (biomechanics, muscle activity)
Almonroeder [21] *	45 healthy females (age: 18 to 25 years) currently/ previously competing in landing/cutting sports at least at recreational level; cross-sectional design	*Single-leg landing with anticipated and unanticipated side-cutting:* Forward jump (1.5 m) from standing position on force plates with subsequent maneuvers: lateral cut on non-dominant limb, single-leg landing on non-dominant limb or bilateral landing and vertical jump; only cutting analyzed *Conditions:* (1) Anticipated (landing maneuver known before jump) (2) Unanticipated (visual stimulus indicating required maneuver displayed only during the jump) - Available response time: minimum of 350 ms - Visual stimulus: illuminating arrows (left, right or vertical) - Stimulus location: 1 m in front of participants (slightly below eye level) - Floor/target visible: yes	*Biomechanics:* 3D kinematics and kinetics and stance time (between landing and cutting) of non-dominant limb, *Cognitive function:* ImPACT (computerized) reaction time test (processing speed)	Slow (>0.59 s) vs. fast (<0.52 s) reaction time group based on impact reaction time test. 2 × 2 ANOVA (group, condition)
Giesche [10]	20 healthy males (age: 27 ± 4 years, BMI: 25 ± 3), physically active (at least at recreational level); cross-sectional design	*Jump with anticipated and unanticipated single-leg landing* Counter-movement jump on capacitive pressure plate (both limbs assessed) with (1) left foot landing or (2) right foot landing *Conditions:* (1) Anticipated (required landing side known before jump) (2) Unanticipated (visual stimulus indicating required landing side displayed only during jump) - Available response time: ∼360 ms - Visual stimulus: footprint sign (left or right) - Stimulus location: 2 m in front of participants (eye level) - Floor/target visible: yes	*Biomechanics:* pVGRF, landing postures (COP path length, TTS, standing errors) Decision-making quality: landing errors (landing on wrong or both sides) in unplanned trials *Cognitive function:* computerized and pen-and-paper tests (TMT-A, B); Stroop I, II, III; digit spans test; stop-signal task; reaction time/processing speed via CogState test battery to capture higher level (cognitive flexibility, working memory, inhibitory and interference control) and lower-level (visual perception, simple reaction time, short-term memory) cognitive functions	Between-condition differences (biomechanics, decision-making quality) to detect unplanned landing costs (significantly decremental landing stability relative to planned trials); Association between individual cognitive functions and unanticipated landing costs
Niederer [11]	18 healthy, physically active participants (8 males; age: 25 ± 2 years, weight: 68 ± 10 kg) included; crossover design	*Unanticipated single-leg landing* Counter-movement jump on capacitive pressure plate (both limbs assessed) with (1) left foot landing or (2) right foot landing (visual stimulus indicating required landing side displayed only during jump); - Available response time: not reported - Visual stimulus: footprint sign (left or right) - Stimulus location: in front of participants (eye level) - Floor/target visible: yes	*Biomechanics/landing success:* pVGRF, landing postures (TTS, landing and standing errors) *Cognitive function:* pen-and-paper tests (TMT-A, B); outcomes assessed after either a functional, classic or control warm-up (movie) protocol pre- and post-neuromuscular fatigue protocol.	3 (warm-up) × 2 (pre- to post-fatigue ANCOVA (covariates; baseline values and fatigue jump times) or pre to post changes via Friedman testing **

CRI = Concussion Resolution Index, LP = low performance group, HP = high performance group, SDMT = the Symbol Digit Modalities Test, MVS = maximum voluntary contraction, HAM = hamstrings, QUAD = quadriceps, CCR = co-contraction ratio, pre-IC = before initial contact, post-IC = first 50 ms after initial contact, pVGRF = peak vertical ground reaction force, COP = center of pressure path length, TTS = time to stabilization, TMT = Trail-Making Test, Stroop I = read words, Stroop II = name colors, Stroop III = word-color interference test; * PhD thesis. ** The original data were provided by Niederer et al. on request. Based on this, we conducted the statistical analyses regarding the potential relationships between unanticipated landing biomechanics/success and cognitive function ourselves using the control warm-up condition (movie) pre-fatigue only.

**Table 2:** The adapted checklist used for the scoring of methodological quality/risk of bias.

Item	Scoring Criteria
**Reporting**	
Aim	The objective of the study is clearly described.
Outcomes	Outcome measures are stated in the Introduction or Methods section. Reliability/validity data are provided. Scored “0” if methods are first mentioned in the Results section.
Sample characteristics	Characteristics of the included participants (e.g., age, sex, body weight/height, sports and performance level) described. Inclusion and exclusion criteria should be stated.
*Motor task/conditions*	The motor task(s) is/are sufficiently described to allow experimental replication.
Confounders	Potential confounders (i.e., assessment of dominant/non-dominant, sex, available response time to react to the visual cue) are listed.
Findings	Adequate and comprehensible reporting of the study findings. All tests mentioned in the Methods section are addressed.
Variability estimates	Standard deviations, standard errors or confidence intervals reported. For non-normally distributed data, the interquartile range is reported.
Actual *p*-values	Actual *p*-values reported instead of the mere reporting of thresholds (e.g., *p* < 0.05).
*Funding*	External funding/grants reported.
**External validity**	
Participant representativeness	The study identified the source and target population and provided sufficient details about related characteristics (e.g., sex, age, activity/performance level or playing position), and these participants were actually included. For example, scored “0” if only males/females were included and this was not mentioned in the objectives, or if elite athletes were included and the objective was not specific for this.
Setting representativeness	The athletic tasks consisted of movements performed in the sports habitually performed by the participants (e.g., cutting/jump-landing in team sports) and contained a clear decision-making component.
**Internal validity**	
Data dredging	N/A: study was identical to pre-registration (if available), or unspecified/unplanned analyses were performed.
Adequate statistics	Adequate inference statistical analyses were applied to answer the research question. Alpha error inflation is controlled for (statistical power is rated as a separate item).
**Internal validity—confounding (selection bias)**	
Accurate measurements	Objective measurement tools with sufficient test quality (reliability/validity) were used.
*Randomness of conditions/directions*	The order of the pre- and unplanned conditions and landing side/cutting direction was randomized.
Adjustment for confounders	Potential confounders were considered as covariates in the statistical analysis, or, for example, it was made clear that potential confounding variables (e.g., approach speed and available response time) did not differ between conditions.
**Sufficient Power**	An a priori sample size calculation was performed and detailed in the Methods section.

Modified or new items are marked in *italics*.

**Table 3:** Study quality (adapted Black and Down checklist).

Item	Herman et al. [19]	Shibata et al. [20]	Almonroeder * et al. [21]	Giesche et al. [10]	Niederer et al. [11]
Aim	1	1	1	1	1
Outcomes	1	1	1	1	1
Sample	1	1	1	1	1
Motor task/conditions	1	1	1	1	1
Confounders	1	1	1	1	1
Findings	1	1	1	1	1
Variability estimates	1	1	1	1	1
Actual *p*-values	1	1	1	1	0
**Total reporting**	**8/8**	**8/8**	**8/8**	**8/8**	**7/8**
Participant representativeness	0	0	0	0	0
Setting representativeness	1	1	1	1	1
**Total external validity**	**1/2**	**1/2**	**1/2**	**1/2**	**1/2**
Data dredging	1	1	1	1	1
Adequate statistics	0	0	1	0	1
**Total internal validity**	**1/2**	**1/2**	**2/2**	**1/2**	**2/2**
Accurate measurements	1	1	1	1	1
Randomization of conditions	1	1	1	1	1
Adjustment for confounders	0	1	1	0	0
**Total internal validity—confounding**	**2/3**	**3/3**	**3/3**	**2/3**	**2/3**
Sufficient power	1	0	1	0	0
**Total power**	**1/1**	**0/1**	**1/1**	**0/1**	**0/1**
**Sum score**	**13/16**	**13/16**	**15/16**	**12/16**	**12/16**

* Ph.D. thesis.

**Table 4:** Synthesized results for the relationship between perceptual–cognitive function and unplanned task safety.

Study	Biomechanical Task Safety	Functional Task Safety
Herman et al. [19]	↓	
Shibata et al. [20]	↓	
Almonroeder et al. [21]	-	
Giesche et al. [10]	↓	?
Niederer et al. [11]	?	↓
Rating of evidence	conflicting	conflicting

Gray-shaded fields: parameter not examined, ↓ = decrease in parameter, - = no difference in parameter, ? = conflicting results. Columns show summarized outcomes grouped as biomechanical (e.g., joint moments/angles) and functional task safety (e.g., success in decision-making or valid trials in jump landings).
